# Protein S-Palmitoylation as Potential Therapeutic Target for Dermatoses

**DOI:** 10.3390/biom16010053

**Published:** 2025-12-30

**Authors:** Yanhai Feng, Jianxin Wu, Hui Tang, Shunying Liu, Honglin Jia, Yi Liang, Zhenglin Li, Lingbo Li, Lingfei Li, Xia Lei

**Affiliations:** 1 Department of Dermatology, Daping Hospital, Army Medical University, Chongqing 400042, China; fengyanhai2025@tmmu.edu.cn (Y.F.); cogitowujx@tmmu.edu.cn (J.W.); xiaohuitang@tmmu.edu.cn (H.T.); sliu2024@tmmu.edu.cn (S.L.); honglinjia@tmmu.edu.cn (H.J.); 15683417308@tmmu.edu.cn (Y.L.); zhenglinli@tmmu.edu.cn (Z.L.); lingboli@tmmu.edu.cn (L.L.); 2 Army 953 Hospital, Shigatse Branch of Xinqiao Hospital, Army Medical University, Shigatse 857000, China; 3 Research Center for Skin Tissue Engineering of Chongqing Higher Education Institutions, Daping Hospital, Army Medical University, Chongqing 400042, China; 4 Air Force Hospital of Chinese PLA Central Theater Command, 589 Yunzhong Road, Pingcheng District, Datong 037006, China

**Keywords:** S-palmitoylation, zDHHCs, dermatoses

## Abstract

Protein S-palmitoylation is a pivotal yet poorly integrated research field in dermatology. This reversible post-translational lipid modification primarily occurs on cysteine residues and is principally catalyzed by zinc finger and Asp-His-His-Cys DHHC-domain containing proteins (zDHHCs). The S-palmitoylation/depalmitoylation cycle directly affects protein localization, trafficking, stability, and protein–protein interaction, thereby regulating a variety of signaling pathways, including those mediating inflammation and immune reaction. Accumulating evidence has indicated that S-palmitoylation regulates various skin biological functions, including skin inflammation, skin barrier function, hair growth, and melanin synthesis, and is ultimately implicated in the initiation and development of massive dermatoses, such as alopecia and psoriasis. The recent development of new research tools, coupled with S-palmitoylation’s therapeutic potential, makes the timely synthesis of its role in skin pathophysiology both critical and opportune. Here, we summarize recent advances in understanding the mechanistic roles of S-palmitoylation in dermatological conditions and evaluate its potential as a therapeutic target for innovative treatment strategies.

## 1. Introduction

For proteins to function adequately in a context-dependent manner, their activity and localization must be tightly regulated in both time and space. Post-translational modifications (PTMs) represent a fundamental mechanism for precisely modulating the physicochemical properties of proteins, thereby directly influencing their functions. Hundreds of distinct PTMs impacting thousands of proteins have been identified in mammalian cells [1,2,3]. Among these, protein lipidation, the covalent attachment of lipid moieties to proteins, constitutes a major class of PTMs [4]. Lipidation confers hydrophobicity upon proteins, thereby promoting their association with cellular membranes and specific membrane subdomains [4,5,6]. Consequently, lipid modifications are essential for establishing the compartmentalized organization of eukaryotic cells and for facilitating coordinated communication between these compartments [5]. Lipid modifications include several major forms, such as N-myristoylation, N-acylation, O-acylation and S-acylation [4,6]. Among these, S-acylation, commonly referred to as S-palmitoylation, has been extensively studied in recent years, with growing emphasis on its roles in skin biology and diseases.

S-palmitoylation is defined as the covalent attachment of a 16-carbon saturated fatty acid (palmitate) to specific cysteine residues of target proteins via a thioester linkage, although other fatty acyl chains (e.g., myristate or oleate) can also be incorporated [7]. Unlike other forms of protein lipidation, S-palmitoylation is uniquely reversible, a property conferred by the labile thioester bond. This reversibility enables rapid, spatiotemporal regulation of protein function, analogous to mechanisms observed in phosphorylation [8]. The S-palmitoylation/depalmitoylation cycle is catalyzed by specific enzymes, which serve as molecular switches of protein function in a manner similar to ubiquitination or phosphorylation [9]. Functionally, S-palmitoylation enhances protein hydrophobicity and is critically involved in modulating protein stability and function, subcellular localization, interaction networks, and signal transduction [10]. Consequently, dysregulation of protein S-palmitoylation is increasingly recognized as an underlying mechanism in the pathogenesis of numerous human diseases [10]. In dermatology, a growing body of literature has established that S-palmitoylation is involved in the occurrence and development of a wide spectrum of skin conditions, including skin barrier conditions, melanogenesis, and hair growth conditions (alopecia), and inflammatory-mediated diseases such as dermatitis and psoriasis [11,12,13,14,15].

Although its fundamental importance is well recognized, the current understanding about S-palmitoylation’s roles in dermatology remains highly fragmented. This fragmentation hinders a system-level understanding of S-palmitoylation as a central regulatory node in skin pathophysiology. The pressing need to bridge this knowledge gap is underscored by the considerable therapeutic potential of targeting the palmitoylation machinery. The emergence of more specific palmitoyltransferase inhibitors, coupled with advances in detection methodologies, has positioned palmitoylation modulation as a promising frontier for drug discovery. Therefore, this review is timely and warranted. In this review, we aim to synthesize current evidence and elucidate the mechanistic underpinnings of palmitoylation in various dermatoses, with a particular focus on its specific associations with skin diseases. We will begin by delineating the fundamental regulatory mechanisms of protein S-palmitoylation. This will be followed by a critical synthesis of recent advances linking S-palmitoylation to specific dermatoses, including alopecia and psoriasis. Furthermore, to provide mechanistic depth, we will examine how S-palmitoylation influences key cellular processes—such as autophagy and ferroptosis—that underpin skin disease pathogenesis. By presenting this comprehensive framework, we seek to catalyze future research and inspire novel therapeutic strategies for a range of difficult-to-treat skin diseases.

## 2. Protein S-Palmitoylation and Its Catalytic Enzyme

**Figure 1:**
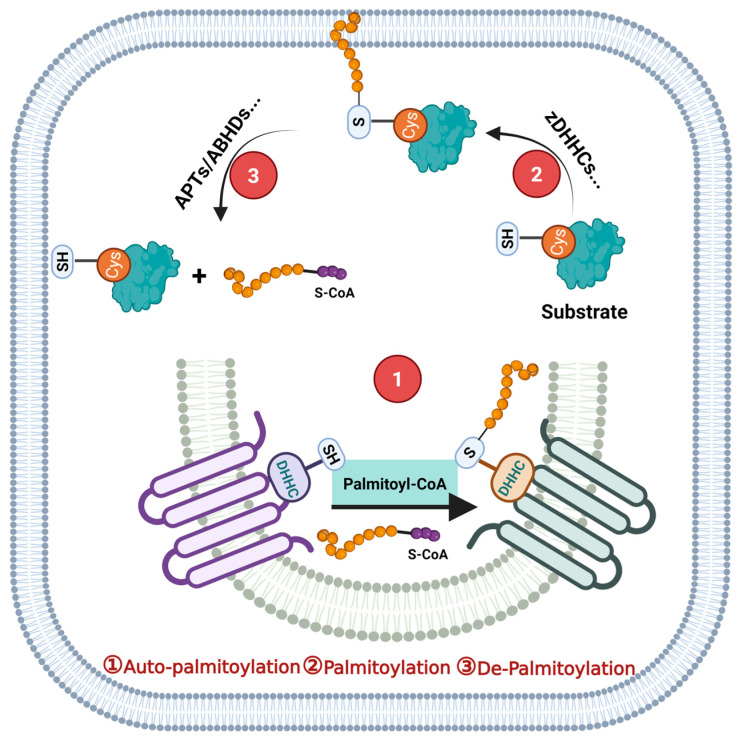
The dynamic regulation of protein S-palmitoylation. ① Palmitoyl acyltransferases perform autopalmitoylation within their DHHC domain in the endoplasmic reticulum or Golgi lumen/plasma membrane. ② The autopalmitoylated palmitoyl acyltransferases transfer palmitate to the substrate cysteines to accomplish palmitoylation. ③ Thioesterases (APTs or ABHDs) remove the palmitate from the palmitoylated proteins, and this process is named depalmitoylation. DHHC: Asp-His-His-Cys, SH: Cys, APT: acyl-protein thioesterases, ABHD: α/β-hydrolase domain, S-palmitoy-coenzyme A (S-CoA). This figure was created in Biorender Feng, Y.H. (2025) https://app.biorender.com/ (accessed on 17 November 2024).

Currently, several hundred mammalian proteins, many of which are implicated in dermatosis, have been identified as substrates for S-palmitoylation (Figure 1). While the majority of palmitoylated proteins contain a single modification site, examples of proteins with as many as five or six individual palmitoylated cysteine residues are known [16]. Notably, certain proteins can undergo autopalmitoylation without enzymatic catalysis [17]. Although various modes of protein S-palmitoylation have been described, the majority are catalyzed by specific palmitoyl S-acyltransferases (PATs) which are characterized by a zinc finger and DHHC domain (zDHHC). The DHHC domain is a cysteine-rich structural motif which contains the conserved Asp-His-His-Cys (DHHC) tetrapeptide sequence [18]. From the perspective of chemical structure, members of the zDHHC family contain at least four transmembrane domains, with the cytoplasmic DHHC cysteine-rich domain located between transmembrane domains 2 and 3 [18]. In mammals, the zDHHC family comprises 23 distinct members which are encoded by 23 different genes, designated as zDHHC 1−24, with the exception of zDHHC10 [10]. Beyond the core DHHC domain, zDHHC family proteins harbor other conserved motifs, including aspartate-proline-glycine (DPG), threonine-threonine-x-glutamic acid (TTxE) and a palmitoyltransferase conserved carboxy-terminal (PaCCT) motif [18]. The differences between zDHHC family proteins not only lie in the N- and C- terminal, but also exist in the overall length and specific sequence composition [18]. Additionally, some zDHHC members contain other well-characterized protein interaction motifs, such as ankyrin repeats in zDHHC13 and zDHHC17, an SH3 domain in zDHHC6, and PDZ-binding motifs in zDHHC5 and zDHHC8 [18]. Thus, zDHHC family proteins possess biological functions beyond their S-palmitoylation-catalyzing abilities, and their roles in dermatological diseases likely represent the integrated actions of these diverse domains.

As transmembrane proteins, zDHHC enzymes are intrinsically localized to various membrane-bound organelles. The majority of zDHHC proteins reside in the endoplasmic reticulum (e.g., zDHHC1, 4, 6, 7) and Golgi apparatus (e.g., zDHHC3, 20) [19,20]. Consequently, the endoplasmic reticulum and Golgi apparatus serve as the primary sites of protein palmitoylation within mammalian cells [21,22,23]. However, several zDHHCs are also present at other locations, including the nuclear envelope (zDHHC6), throughout the early secretory pathway (e.g., zDHHC2, 4, 14, 19, 21) [23,24,25], and within the endocytic system (zDHHC2, 5) [18]. Thus, membrane-bound organelles should be the potential targets when zDHHCs regulate dermatosis. Defining the specific organelles involved is therefore critical for a deeper understanding of S-palmitoylation’s role in dermatological diseases. In addition, the subcellular localization of zDHHCs is dynamic, implying that their roles in dermatosis cannot be fully understood from a static perspective.

These zDHHC enzymes catalyze protein S-palmitoylation via a two-step enzymatic mechanism. The initial step involves autoacylation of the conserved cysteine residue within the DHHC motif [25]. Specifically, this cysteine residue within the active site undergoes acylation through reaction with an acyl-coenzyme A (acyl-CoA) substrate, which binds to a conserved CoA-binding surface on the cytosolic side of the enzymes [26]. In the second step, the acyl chain is transferred from the enzyme to a cysteine residue on the target protein substrate [27]. Beyond this canonical two-step mechanism, additional modes of protein S-palmitoylation regulation have been discovered. For example, the Golgi-resident S-palmitoylation machinery comprises multiple zDHHC enzymes with major differences in substrate specificities and S-palmitoylation efficiencies [28]. Different catalyzing pathways may occur under diverse conditions and lead to entirely different results. Therefore, elucidating these context-dependent regulatory mechanisms may provide mechanistic insights into the less characterized functions of S-palmitoylation. Although the thioester linkage in protein S-palmitoylation is generally hydrolytically stable, it can be cleaved by various acyl-protein thioesterases. This hydrolysis process is termed depalmitoylation. While acyl-protein thioesterases (APTs) 1/2 (APT 1/2, LYPLA1/2) are the most characterized depalmitoylating enzymes, other hydrolases, including ABHD12, ABHD13 and ABHD17, have been identified more recently [8]. Key evidence linking specific palmitoylated proteins, PATs, and APTs to skin biological function and dermatosis is summarized. Therefore, targeting the proteins which are S-palmitoylated and intervening with specific zDHHC enzymes are the basic strategies for managing skin-associated diseases.

## 3. Protein S-Palmitoylation Impacts Skin Physiological Processes and Dermatosis

**Figure 2:**
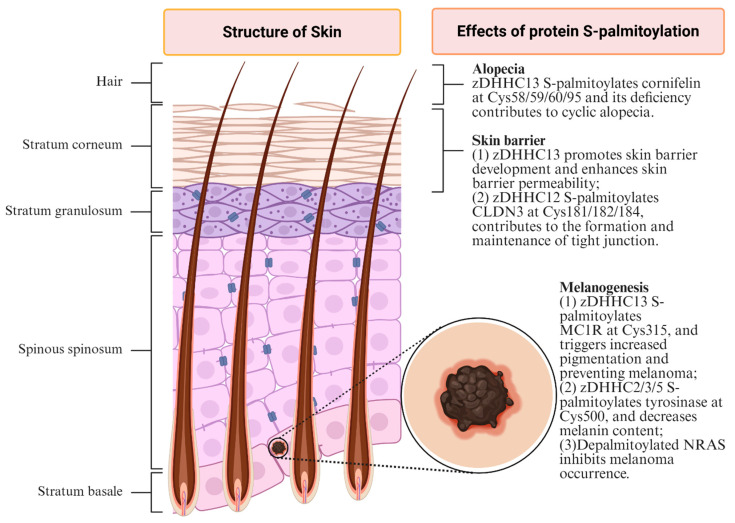
Effects of protein S-palmitoylation on main dermatosis. (1) Alopecia: zDHHC13 S-palmitoylates cornifelin at Cys58/59/60/95 and its deficiency contributes to cyclic alopecia [13,29]. (2) Skin barrier: zDHHC13 promotes skin barrier development and enhances skin barrier permeability [14,30]; zDHHC12 S-palmitoylates CLDN3 at Cys181/182/184, contributes to the formation and maintenance of tight junction [29]. (3) Melanogenesis: zDHHC13 S-palmitoylates MC1R at Cys315, and triggers increased pigmentation and preventing melanoma [31]; zDHHC2/3/5 S-palmitoylates tyrosinase at Cys500, and decreases melanin content [15]; Depalmitoylated NRAS inhibits melanoma occurrence [32]. This figure was created in Biorender Feng, Y.H. (2025) https://app.biorender.com/ (accessed on 17 November 2024).

Protein S-palmitoylation contributes to the pathogenesis of multiple dermatological disorders by modulating key pathological processes. A clear understanding of the mechanistic roles of S-palmitoylation in these skin diseases is therefore essential. As summarized in Figure 2, S-palmitoylation has been implicated in a spectrum of dermatological conditions, including alopecia, skin barrier conditions, melanogenesis, inflammatory-related diseases, and carcinogenesis.

### 3.1. Skin Barrier

The skin barrier serves as the body’s primary defense, and its impairment is a major contributor to numerous dermatological conditions. Emerging evidence indicates that protein palmitoylation is critically involved in the skin barrier. Chen et al. demonstrated that deficiency of palmitoyl-acyl transferase zDHHC13 impairs skin barrier development and increases permeability, rendering mice susceptible to environmental bacterial infection and inflammatory dermatitis [14,30]. Furthermore, as tight junctions are core components of the skin barrier, Yuan et al. discovered that claudin-3 (CLDN3), a protein critical for the formation and maintenance of tight junctions, is S-palmitoylated at Cys181, Cys182, and Cys 184 by zDHHC12 [29]. This S-palmitoylation event promotes the correct plasma membrane localization of claudin-3 and is essential for skin barrier integrity [29]. Thus, protein S-palmitoylation mediated by different zDHHC family proteins can exert divergent effects on skin barrier regulation, primarily determined by their specific protein substrates. Therefore, elucidating the precise roles of S-palmitoylation in skin barrier function requires a clear identification of the specific substrates involved.

### 3.2. Melanogenesis

Melanin is a key skin pigment whose dysregulation underlies several pathological conditions, including melanoma. In mammals, melanin is synthesized within melanocytes by specialized organelles termed melanosomes. These pigment-laden melanosomes are subsequently transferred to keratinocytes, thereby determining visible pigmentation in animal’s hair and skin [32]. The melanocortin-1 receptor (MC1R), one major regulating molecule of human and mouse pigmentation, is S-palmitoylated at Cys315, mediated by zDHHC13 [31]. This modification activates MC1R signaling, enhances pigmentation, and confers protection against melanoma development [31]. Meanwhile, tyrosinase, the rate-limiting enzyme in melanin synthesis, is S-palmitoylated by zDHHC2, 3 and 5 at Cys500, which promotes its ubiquitination and subsequent degradation, ultimately reducing melanin content [15]. Melanoregulin, encoded by the dilute suppressor gene, has been implicated in the regulation of melanosome transport within mammalian epidermal melanocytes [29]. Wu et al. demonstrated that melanoregulin, which negatively regulates melanosome transfer from melanocytes to keratinocytes, is N-terminally S-palmitoylated; this modification stabilizes melanoregulin on the melanosome membrane and ensures proper transfer [32]. In melanoma, oncogenic NRAS, whose mutations are major drivers of this disease, undergoes a continuous S-palmitoylation/depalmitoylation cycle [32]. Disruption of this cycle by AP-1 or AP-2 perturbs neuroblastoma rat sarcoma viral oncogene homolog (NRAS) membrane localization, leading to abnormal cell proliferation and melanoma pathogenesis [31]. Collectively, these findings underscore the critical importance of protein S-palmitoylation in regulating melanin production and melanin-associated dermatosis, particularly melanoma. Nevertheless, research in this field remains limited. Future studies should prioritize elucidating the organelle-specific mechanisms of S-palmitoylation in melanocyte biology.

### 3.3. Skin Virus Infection

Viral infections, particularly human papillomavirus (HPV) infection, contribute to several dermatoses with high prevalence, such as verruca vulgaris. Therefore, identifying effective measures for prevention and treatment of these viral skin diseases is of considerable significance. Emerging research has revealed a crucial role for S-palmitoylation in antiviral immunity, particularly against RNA viruses. Among key antiviral effectors, interferon-induced transmembrane proteins (IFITM) are non-negligible [33], and IFITM3 is S-palmitoylated at Cys71, Cys72, and Cys105, which strengthens its antiviral capability [34]. Specifically, S-palmitoylation of IFITM3 at Cys72 modulates its conformation and membrane interactions, thereby enhancing its capacity to engage incoming viral particles, block their cytoplasmic entry, and accelerate their lysosomal clearance [35]. In addition, Liu et al. have discovered that mitochondrial antiviral signaling protein (MAVS), a C-terminal tail-anchored mitochondrial outer membrane protein, is S-palmitoylated by zDHHC7 at Cys508 [35]. This modification stabilizes MAVS aggregates on the mitochondrial outer membrane and promotes downstream antiviral signal transduction [36]. The above evidence highlights that protein S-palmitoylation constitutes an essential host defense against cutaneous viral infections. Nevertheless, further research is needed to identify additional S-palmitoylated antiviral proteins and evaluate their potential as therapeutic targets.

### 3.4. Alopecia

Hair abnormalities, including alopecia, represent common dermatological conditions whose pathogenesis and treatment strategies remain challenging clinical issues. Previously, zDHHC13 has been reported to be the guard of hair growth through its palmitoyltransferase activity [13]. Consistent with this role, Liu et al. further identified that zDHHC13 deficiency contributed to ragged and dilapidated cuticles of the hair shaft, poor hair anchoring ability and premature hair loss at the early telogen phase of the hair cycle, collectively leading to cyclic alopecia [30]. Additionally, zDHHC13-deficient mice exhibit epidermal hyperproliferation, disturbed cornification, and a fragile cornified envelope [30]. Further investigation unveiled that cornifelin is S-palmitoylated by zDHHC13 at Cys58, Cys59, Cys60, and Cys95 [30]. Thus, zDHHC13 deficiency has been established as a significant etiological factor in hair abnormalities. More importantly, these studies have broadened the fields of uncovering the mechanisms of alopecia. Nevertheless, other zDHHC family proteins and corresponding pathways should be the next focuses.

### 3.5. Atopic Dermatitis, Psoriasis and Other Skin Inflammatory Processes

**Figure 3:**
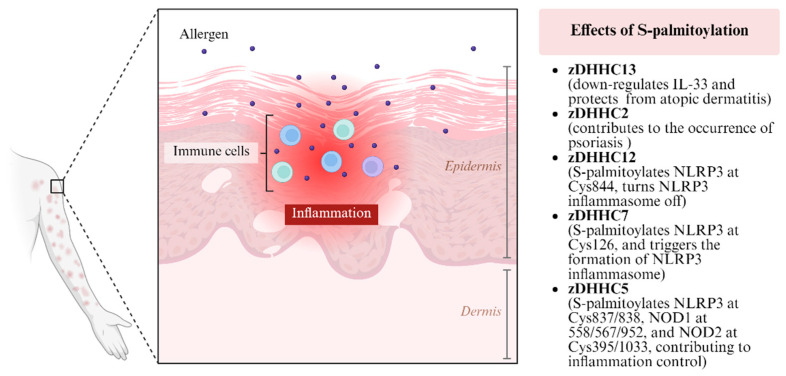
Effects of protein S-palmitoylation on inflammatory dermatosis. zDHHC 13 [11], zDHHC 2 [12], zDHHC 12 [37], zDHHC 7 [31], and zDHHC 5 [33,34,35] have been verified to impact inflammatory dermatosis through different pathways. This figure was created in Biorender Feng, Y.H. (2025) https://app.biorender.com/ (accessed on 17 November 2024).

Inflammation is a central driver in the pathogenesis of various dermatological conditions, including psoriasis and atopic dermatitis. Therefore, elucidating the mechanisms of skin inflammation remains a major research focus, with S-palmitoylation emerging as a key regulatory process of interest (Figure 3). Innate immunity is known to drive the initiation and progression of inflammatory dermatosis, including psoriasis and atopic dermatitis. Chen Li-Ying et al. have reported that mice with epidermal-specific deletion of zDHHC13, a key S-palmitoylation enzyme, are susceptible to environmental bacteria and exhibit persistent skin inflammation [11]. This phenotype is characterized by elevated interleukin-33 (IL-33) and type 2 innate lymphoid cells, culminating in an atopic dermatitis-like disease [11]. Furthermore, zDHHC2 has been found to be robustly upregulated in psoriatic skin, and its genetic ablation dramatically inhibited the pathology in a mouse model of psoriasis [12]. These findings illustrate that different zDHHC proteins can exhibit diverse roles in inflammatory dermatosis. A deeper understanding of specific innate immune pathways is needed to fully delineate the precise roles of S-palmitoylation in these diseases. To this end, we focus on two key innate immune sensors: the nucleotide-binding oligomerization domain (NOD)-like receptor family pyrin-domain containing 3 (NLRP3) and NOD1/2. NLRP3 is a critical pattern recognition receptor in innate immunity that detects a wide range of pathogen-associated and damage-associated molecular patterns [29]. Recently, Wang Liqiu et al. demonstrated that NLRP3 S-palmitoylation at Cys844 mediated by zDHHC12 promotes its degradation through chaperone-mediated autophagy, turning the NLRP3 inflammasome off [37]. Conversely, impaired NLRP3 S-palmitoylation predisposes one to the occurrence of inflammatory diseases, such as lipopolysaccharide (LPS)-induced endotoxic shock [37]. Beyond Cys844, Yu Tao et al. has further identified Cys126 as another palmitoylation site on NLRP3, catalyzed by zDHHC7 in macrophages [31]. This modification is essential for trans-golgi network (TGN) localization of NLRP3, facilitating ASC recruitment and inflammasome assembly [31]. Moreover, zDHHC5 mediates NLRP3 palmitoylation at Cys837/838, which is required for NLRP3-NEK7 interaction and subsequent inflammasome activation [33]. This modification is dynamically reversed by the depalmitoylase ABHD17A [33]. Collectively, this evidence establishes the NLRP3 inflammasome as a key substrate regulated by S-palmitoylation. We next turn to NOD1 and NOD2, additional key sensors of the innate immune system. zDHHC5 palmitoylates NOD1 at Cys558, Cys567, and Cys952, and NOD2 at Cys395 and Cys1033 [34,35]. These modifications enhance receptor stability by attenuating their autophagic degradation, thereby potentiating bacterial sensing and pro-inflammatory signaling [34,35]. Together, S-palmitoylation mediated by ZDHH5, 7, and 12 critically influences innate immunity system sensors such as NLRP3 and NOD1/2. This highlights the versatile, and often opposing, regulatory potential of S-palmitoylation and unveils the huge research opportunities in this field. Future studies should aim to elucidate the role of S-palmitoylation in other innate immune pathways, such as the AIM2 inflammasome, which is also implicated in the pathogenesis of inflammatory skin diseases.

### 3.6. Skin Carcinogenesis

Skin carcinogenesis is a common malignancy, yet its underlying pathogenesis remains incompletely understood. Therefore, unearthing the pathogenic mechanisms is crucial for advancing our understanding and facilitating the development of effective targeted therapies. Concerning this field, Perez et al. described a spontaneous mutation in the *zDHHC13* gene that underlies a spectrum of phenotypes, including generalized hypotrichsis development, disrupted hair cycle, epidermal and sebaceous gland hyperplasia, hyperkeratosis, and increased epidermal thickness [38]. In addition, *zDHHC13*-mutant mice exhibit significantly increased tumor multiplicity and accelerated malignant progression of papillomas following chemical skin carcinogenesis, indicating a protective role for zDHHC13 against skin carcinogenesis [38]. However, only zDHHC13 has been verified to affect the occurrence and progression of skin carcinogenesis; future research should prioritize investigating the roles of other zDHHC enzymes and S-palmitoylation events in skin tumorigenesis.

Collectively, zDHHC members are critically involved in diverse dermatologic phenotypes, including skin barrier development, melanogenesis, alopecia, atopic dermatitis, psoriasis, skin inflammatory, and carcinogenesis (Table 1). Among these, zDHHC13 stands out as a central regulator, with its activity implicated in a wide spectrum of skin physiology and pathology. It acts as a crucial positive regulator in skin biology, fostering skin barrier formation, melanogenesis, and hair growth. Conversely, its deficiency predisposes one to a range of pathologies, including alopecia, carcinogenesis, and atopic dermatitis. In the context of skin inflammatory diseases such as atopic dermatitis, zDHHC5 has emerged as a particularly significant regulator. It palmitoylates key innate immune molecules such as NLRP3, NOD1, and NOD2, thereby shaping the development and progression of these conditions. Taken together, these findings underscore that individual zDHHC enzymes, such as the multi-functional zDHHC13 and the inflammation-specific zDHHC5, operate in a coordinated manner to govern overall skin homeostasis and disease.

## 4. Protein S-Palmitoylation Exerts Roles Through Impacting Cellular or Physiological Processes

**Figure 4:**
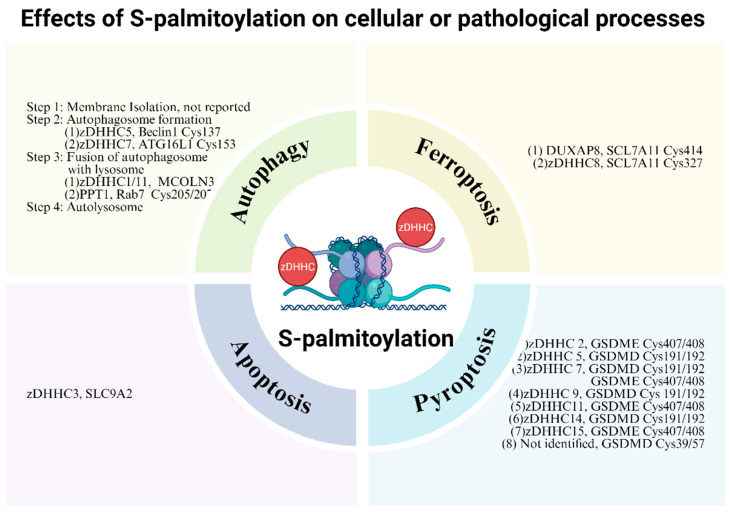
Effects and corresponding substrate molecules of S-palmitoylation on cellular or pathological processes including autophagy [39,40,41,42,43], ferroptosis [44,45], apoptosis [46], and pyroptosis [47,48,49,50,51,52,53,54]. This figure was created in Biorender Feng, Y.H. (2025) https://app.biorender.com/ (accessed on 17 November 2024).

S-palmitoylation is an essential regulator of diverse cellular processes. Accumulating evidence now positions zDHHC enzymes as promising therapeutic targets for various diseases, owing to their pivotal roles in critical processes, including cellular differentiation, autophagy, pyroptosis, ferroptosis, and apoptosis (Figure 4). This section will therefore focus on the influence of S-palmitoylation on cellular or pathological processes.

### 4.1. Cellular Differentiation

Cellular differentiation is a fundamental process in skin biology, and its abnormality underlies the pathogenesis of several dermatoses, including psoriasis and Olmsted syndrome. Therefore, elucidating the molecular mechanisms governing aberrant cellular differentiation is crucial for understanding these skin disorders. Enabled by technological advances, global studies have established S-palmitoylation as an important and pervasive post-translational modification in eukaryotes with the capacity to coordinate diverse biological processes [8]. As a principal biological process, cellular differentiation is recognized to be tightly regulated by S-palmitoylation. Evidence from *S. prombe* and *Cryptococcus neoformans* supports that S-palmitoylation affects sexual differentiation of eukaryotes through distinct signaling pathways [8]. In *S. prombe*, the newly identified PAT Erf2 catalyzes palmitoylations of Rat Sarcoma viral oncogene homolog (Ras1), Ras homology 2 (Rho2), and Ras homology 3 (Rho3) [8]. These modifications are critical for plasma membrane morphogenesis, meiotic division, and cell wall integrity maintenance, respectively [8]. In *Cryptococcus neoformans*, the DHHC-PAT-encoding gene palmitoyltransferase 4 (*PFA4)* has dramatic effects on its morphology, stress tolerance and virulence potential [38]. Comparative palmitoyl proteome profiling identified that PFA4 substrates are involved in cell wall synthesis, membrane transport, signal transduction, and membrane trafficking [55]. Furthermore, Ras1 palmitoylation mediated by PFA4 is tightly associated with sexual differentiation in *C. neoformans* [56]. Collectively, the above literature has preliminary suggested that protein S-palmitoylation profoundly influences cellular differentiation across eukaryotes. More direct evidence from mouse models shows that zDHHC13^skc4^ mice exhibit dilapidated cuticles of the hair shaft, poor hair anchoring, premature hair loss at the early telogen phase, epidermal hyperproliferation, disturbed cornification, and a fragile cornified envelope, collectively leading to cyclic alopecia and impaired skin barrier function [30]. These findings imply that zDHHC13-mediated protein S-palmitoylation represents a key signaling pathway regulating hair and skin differentiation [30]. In summary, protein S-palmitoylation is intimately linked to cellular differentiation and represents a promising therapeutic target for dermatoses which are characterized by abnormal differentiation. Nevertheless, this field is relatively underinvestigated, and more regulatory pathways are waiting for elucidation.

### 4.2. Autophagy

Multiple skin disorders, including psoriasis, have been closely linked to autophagy. Autophagy is a conserved self-degradative process important for maintaining cellular energy homeostasis during development and under nutrient stress. It also plays a housekeeping role in removing misfolded or aggregated proteins and clearing damaged organelles (e.g., mitochondria, endoplasmic reticulum, and peroxisomes), as well as eliminating intracellular pathogens. From the perspectives of biological processes, autophagy proceeds through four key steps: phagophore nucleation, autophagosome formation, autophagosome–lysosome fusion, and autolysosome degradation [57]. As one principal step, autophagosome formation is initiated by PI3KC3 complex I (class III phosphoinositide 3-kinase, PI3KC3-C1) [58]. This complex, comprising beclin1, PIK3C3, and the adapter proteins autophagy-related protein 14-like (ATG14L) and vacuolar protein sorting 15 (VPS15) [58], is tightly regulated by S-palmitoylation. Specifically, zDHHC5-mediated S-palmitoylation of beclin1 at Cys 137 stimulates the assembly of ATG14L-containing PI3KC3-C1 and facilitates its lipid kinase activity by reinforcing hydrophobic interactions between beclin1 and the adaptors ATG14L and VPS15 [39]. In addition, PI3KC3-C1 activity is modulated by a set of autophagy-related (ATG) proteins [58]. Among ATG proteins, ATG16L1 is particularly prominent for its important roles in catalyzing the lipidation of MAP1LC3/LC3 (microtubule-associated protein 1 light chain 3) and promoting autophagosome formation after forming an ATG12-ATG5-ATG16L1 complex [40]. The biological function of ATG16L1 is, in turn, regulated by S-palmitoylation. A recent study demonstrated that the S-palmitoylation of ATG16L1 at Cys 153, catalyzed by zDHHC7, is essential for its biological function [41]. The Ca^2+^-permeable cation channel-mucolipin 3 (MCOLN3/TRPML3) also regulates autophagosome formation and autophagosome–lysosome fusion in an S-palmitoylation-dependent manner [42]. The S-palmitoylation of MCOLN3/TRPML3 at the C-terminal region (Cys549, Cys550, Cys551) mediated by zDHHC1/11 promotes its intracellular trafficking, triggering robust Ca^2+^ release that promotes and autophagosome–lysosome fusion [42]. Conversely, MCOLN3/TRPML3 depalmitoylation disrupts autophagic flux and abolishes nutrient starvation-induced autophagy [42]. Moreover, the small GTPase Rab7, critical for autophagosome–lysosome fusion, requires S-palmitoylation at Cys205/207 for its trafficking to late endosomal/lysosomal membranes [42]. Uniquely, this process is mediated by palmitoyl-protein thioesterase-1 (PPT1) [43]. Taking these findings together, there is growing interest in the regulation of autophagy by S-palmitoylation, particularly the roles ofzDHHC1, 5, 7, and 11 in autophagosome formation and autophagosome–lysosome fusion. However, a more comprehensive understanding of palmitoylation in autophagy will require focused investigation into other stages (e.g., phagophore elongation) and selective autophagy pathways, such as mitophagy and chaperone-mediated autophagy.

### 4.3. Pyroptosis

Pyroptosis is a lytic form of programmed cell death triggered by proinflammatory signals and executed primarily by pore-forming gasdermins [59]. Among gasdermins, gasdermin D (GSDMD) is the most extensively characterized, with its N-terminal domain (GSDMD-NT) serving as the key effector motif [47]. Recent research has established that S-palmitoylation of GSDMD at Cys 191/192 (human/mouse), catalyzed by zDHHC5, 7, 9, or 14 in a context-dependent manner and facilitated by reactive oxygen species (ROS), directly mediates GSDMD-NT membrane translocation, pore-forming, and subsequent pyroptosis activation and cytokine release [47,48,49,50,51]. Further work conducted by Jiang Xueqin et al. confirmed that inhibiting GSDMD S-palmitoylation at Cys191 (using NU6300 or via APT2-mediated depalmitoylation) impedes GSDMD-NT membrane localization and oligomerization, thereby suppressing pyroptosis [50,52]. Collectively, the above evidence points out that the palmitoylation–depalmitoylation cycle spatiotemporally controls GSDMD activation during pyroptosis. Beyond Cys 192, GSDMD is also palmitoylated at Cys 39/57, which promotes the formation of highly ordered dimers and trimers essential for subsequent membrane targeting and insertion [53]. Similarly, GSDME, another classic pyroptosis mediator, undergoes S-palmitoylation, mediated by zDHHC 2, 7, 11, or 15, which is essential for GSDME-induced pyroptosis [54]. In summary, S-palmitoylation is a major post-translational mechanism regulating gasdermin function and pyroptotic cell death. Although plenty of relevant studies have been conducted, the focus has remained predominantly on GSDMD Cys191/192. Future studies should therefore explore S-palmitoylation of other gasdermin family members and their functional roles, particularly in the context of skin pathophysiology. For example, GSDMA is the most abundant gasdermin in the skin and is vital for skin barrier regeneration [60]; however, its S-palmitoylation and relevant roles in skin-associated diseases remain entirely unexplored.

### 4.4. Ferroptosis

Ferroptosis, an iron-dependent form of programmed cell death, is implicated in the pathogenesis of various skin-associated diseases. This cell death process is driven by the fatal accumulation of lipid peroxides and is tightly regulated by multiple critical factors, notably the cystine/glutamate antiporter solute carrier family 7 member 11 (SCL7A11) [44]. SCL7A11 has recently been confirmed as a substrate for S-palmitoylation. Shi et al. first reported that SCL7A11 could be S-palmitoylated by double homeobox A pseudogene 8 (DUXAP8) at Cys414, delaying its lysosomal degradation, enhancing its bioactivity and suppressing ferroptosis [45]. Subsequently, Wang et al. have broadened this field by demonstrating that zDHHC8-mediated S-palmitoylation of SCL7A11 at Cys327 decreases its ubiquitination level, thereby inhibiting ferroptosis [44]. Collectively, these findings establish S-palmitoylation as an essential element regulating ferroptosis. However, among the numerous proteins regulating ferroptosis, SCL7A11 remains the only one conclusively identified as an S-palmitoylation substrate. Therefore, future research should focus on identifying palmitoylation events on other core ferroptosis regulators, such as glutathione peroxidase 4 (GPX4), and delineating the underlying molecular mechanisms.

### 4.5. Apoptosis

Apoptosis, a form of programmed cell death that typically does not elicit an inflammatory response, is well known to play a significant role in the pathogenesis of a wide spectrum of dermatoses. This process is modulated by numerous proteins, including the sodium/hydrogen exchanger solute carrier family 9 member A2 (SLC9A2). Recent research has reported that SLC9A2 S-palmitoylation is the mechanism of zDHHC3-induced apoptosis [46]. This finding widens the research direction concerning how S-palmitoylation participates in apoptosis. However, caspase family proteins, the principal executioners of apoptosis, have not yet been reported to be S-palmitoylated. Thus, substantial work remains to elucidate the full extent of palmitoylation’s role in apoptotic regulation.

Together, zDHHC members are critically involved in regulating fundamental physiological processes, including cell differentiation, autophagy, pyroptosis, ferroptosis, and apoptosis (Table 2). Protein S-palmitoylation is evidenced to participate in differential steps of these physiological processes. For example, S-palmitoylation of Beclin 1 Cys137 and ATG16L1 Cys153 mediated by zDHHC5 is required for autophagosome formation, whereas PPT1-catalyzed S-palmitoylation of Rab7 at Cys205/207 regulates autolysosome maturation. Furthermore, the regulatory landscape of palmitoylation exhibits considerable complexity, as a single protein substrate can be modified by multiple zDHHCs at distinct, partial-overlapping sites. For instance, GSDMD, the major effector of pyroptosis, could be palmitoylated by zDHHC 5, 7, 9, or 14 at Cys 191/192 or Cys 39/57, respectively. Therefore, zDHHC enzymes constitute a sophisticated regulatory network that fine-tunes critical physiological processes, with mechanisms ranging from the control of sequential steps (e.g., in autophagy) to the multi-enzyme, multi-site regulation of key effector proteins (e.g., GSDMD in pyroptosis).

## 5. Protein S-Palmitoylation Exerts Its Roles Through Impacting Specific Organelles

**Figure 5:**
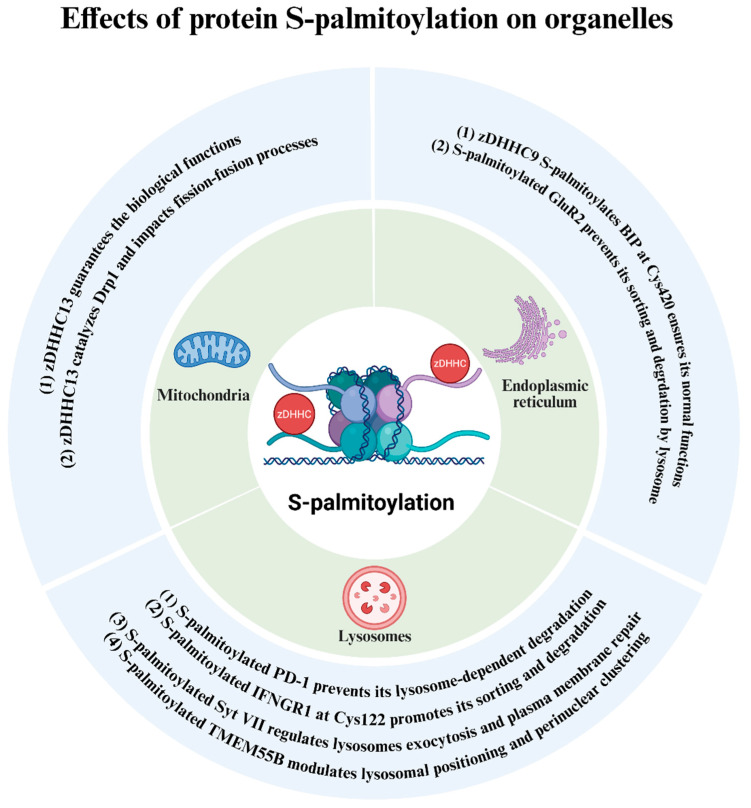
Effects of protein S-palmitoylation on organelles. (1) Mitochondria: zDHHC13 guarantees the biological functions; zDHHC13 catalyzes Drp1 and impacts fission-fusion processes [61,62]. (2) Endoplasmic Reticulum: zDHHC9 S-palmitoylates BIP at Cys420 ensures its normal functions [63]; S-palmitoylated GluR2 prevents its sorting and degrdation by lysosome [64]. (3) Lysosomes: S-palmitoylated TMEM55B modulates lysosomal positioning and perinuclear clustering [65]; S-palmitoylated Syt VII regulates lysosomes exocytosis and plasma membrane repair [66]; S-palmitoylated IFNGR1 at Cys122 promotes its sorting and degradation [67]; S-palmitoylated PD-1 prevents its lysosome-dependent degradation [68]. This figure was created in Biorender Feng, Y.H. (2025) https://app.biorender.com/ (accessed on 17 November 2024).

Organelles serve as the fundamental functional units of cells, and their dysfunction constitutes a key etiological factor in numerous dermatological disorders. Emerging evidence has identified S-palmitoylation as a critical regulator of essential organelles, including mitochondria, endoplasmic reticulum, and lysosomes (Figure 5). This section reviews the current understanding of how S-palmitoylation governs the function of these organelles.

### 5.1. Mitochondria

An aberrant mitochondrial structure or function contributes to diverse cutaneous manifestations, including hair abnormalities, rashes, disordered pigmentation, and acrocyanosis [69]. This insight highlights the therapeutic potential of targeting mitochondrial dysfunction in numerous dermatoses. In this field, zDHHC13, a palmitoyltransferase, has been reported to be essential for maintaining mitochondrial integrity, as its deficiency impairs mitochondrial function [61]. Further studies have confirmed that dynamin-related protein 1 (Drp1), a key regulator of mitochondrial fission–fusion dynamics, has been discovered to be S-palmitoylated by zDHHC13, which is essential for normal fission–fusion cycling [62]. To date, zDHHC13 remains the only palmitoyltransferase definitively linked to the regulation of mitochondrial function. Consequently, this area remains understudied, and future research should concentrate on identifying additional palmitoyltransferases and corresponding mitochondrial substrate proteins, as well as elucidating their roles in specific dermatosis.

### 5.2. Endoplasmic Reticulum

Endoplasmic reticulum stress refers to the aggregation of unfolded or misfolded proteins in the endoplasmic reticulum and a concomitant disruption of calcium ion homeostasis, typically triggered by various exogenous insults [70]. The recent literature has demonstrated a strong link between the endoplasmic reticulum and the pathogenesis of dermatological conditions, including photoaging [70]. S-palmitoylation is crucial for maintaining endoplasmic reticulum homeostasis and modulating endoplasmic reticulum stress responses. The binding immunoglobulin protein (BIP), a principal regulator and biomarker of ER stress, has recently been reported to be S-palmitoylated by zDHHC9 at Cys420, an event associated with decreased endoplasmic reticulum stress [63]. Furthermore, both GluR1 and GluR2, major subunits of the α-amino-3-hydroxy-5-methyl-4-isoxazolepropionic acid receptor (AMPAR), are S-palmitoylated. Intriguingly, diminished S-palmitoylation of GluR2 results in the loss of its mature mode, but leaves GluR1 intact, as S-palmitoylation promotes the sorting of GluR2 to the lysosome and then its degradation [64]. However, research in this field remains relatively limited. Therefore, future studies should prioritize the investigation of other critical ER stress mediators, such as GP73, and elucidate how S-palmitoylation and the corresponding enzymes influence ER structure and function.

### 5.3. Lysosomes

Lysosomes serve as dual-function organelles, acting as both degradation centers and signaling hubs that critically regulate cellular homeostasis, development, and aging. Changes in lysosome function and structure integrity are vital pathological mechanisms of several dermatoses, including psoriasis [71]. Rudnik et al. have demonstrated that S-palmitoylation contributes to the regulation of lysosome function [65]. Specifically, TMEM55B, an integral lysosomal membrane protein, is S-palmitoylated at multiple cysteine residues [71]. Impairment of this modification abolishes TMEM55B’s ability to modulate lysosomal positioning and perinuclear clustering [65]. Beyond TMEM55B, synaptotagmin VII (Syt VII), a Ca^2+^ sensor that regulates lysosome exocytosis and plasma membrane repair, requires S-palmitoylation for its proper function [66]. In addition, accumulating evidence identifies that S-palmitoylation serves as a sorting signal for lysosome degradation, though its functional consequences vary substantially depending on the specific protein substrate. For example, Du et al. discovered that S-palmitoylation of IFN-gamma receptor subunit 1(IFNGR1) at Cys122 is the structural foundation of its sorting and degradation by lysosomes [67]. Conversely, S-palmitoylation of programmed cell death protein 1 (PD-1) promotes its trafficking to the recycling endosome, thereby bypassing lysosome-dependent degradation [68]. Although several S-palmitoylated proteins have been verified to influence lysosome function, the specific palmitoyl S-acyltransferases responsible for these modifications remain largely uncharacterized. Furthermore, the roles of S-palmitoylation on lysosomes structure are required to be elucidated. In summary, S-palmitoylation represents a crucial regulatory layer for lysosomal biology, yet this field remains in its infancy and warrants extensive further investigation.

Thus, zDHHC enzymes critically regulate the function of specific organelles (Table 3). Current research has highlighted a critical and growing emphasis on the roles of S-palmitoylation in lysosomal biology. Although several S-palmitoylated proteins (e.g., TMEM55B, Syt VII, IFNGR1, PD-1) are known to modulate lysosome function, their cognate zDHHC enzymes remain elusive. In summary, despite progress in recognizing palmitoylation’s role in organelles like the lysosome, mapping specific zDHHC enzymes to their physiological substrates represents a major challenge and a significant knowledge gap in the field.

## 6. Great Treatment Potential of Protein S-Palmitoylation Inhibitors in Dermatosis

As discussed above, S-palmitoylation is essential for numerous fundamental physiological processes in skin physiology and pathology, triggering the development of the use of zDHHC enzymes or their corresponding inhibitors as novel drug targets for a range of skin disorders (Table 4). Consequently, developing inhibitors targeting the zDHHC protein family represents a rational therapeutic approach. The most characterized compound in this context is 2-bromopalmitic acid (2-BP), which inhibits S-palmitoylation through forming a covalent bond with the cysteine in the DHHC motif [72]. However, 2-BP lacks selectivity and possesses several drawbacks. Published evidence demonstrates that 2-BP acts as a broad-spectrum, irreversible inhibitor of numerous lipid-metabolizing and membrane-associated enzymes, including mono-, di-, and triacylglycerol acyltransferases, fatty acyl-CoA ligase, and glycerol-3-phosphate acyltransferase, as well as non-lipid-processing enzymes such as NADPH cytochrome-C reductase and glucose-6-phosphatase [68]. Its reactive α-halo-carbonyl moiety indiscriminately alkylates cysteine residues across a wide range of proteins, accounting for the irreversible inhibition of diverse enzymes and highlighting its significant potential for off-target effects [68]. Therefore, the development of selective inhibitors represents a critical next step. Using 2-BP as a starting point, Hong et al. employed acylation-coupled lipophilic induction of polarization (Acy-cLIP) to screen relatively selective zDHHC inhibitors [72]. They identified MY-D-2, MY-D-4, MY-D-5, and MY-D-6 as selective inhibitors of zDHHC3/7, with MY-D-4 exhibiting the highest potency [73]. In a separate approach, Salaun et al. developed and implemented a novel fluorescence resonance energy transfer (FRET)-based high-throughput assay for the discovery of compounds targeting zDHHC2 autoacylation [74]. This screening identified that tetrazole-containing compounds (TTZ-1 and TTZ-2) could inhibit both zDHHC2 autoacylation and its substrate’s S-acylation activity [74]. Thus, only a few inhibitors targeting specific zDHHC proteins have been clarified, and their therapeutic potential in dermatoses is still obscure. This triggers the urgent need to discover additional highly selective inhibitors.

Regarding clinical translation, 2-BP currently represents the most advanced candidate, demonstrating efficacy in cancer models by targeting zDHHC3 and subsequently blocking the programmed cell death-1/programmed cell death-ligand 1 (PD-A/PD-L1) immune checkpoints axis [75]. However, the clinical development of S-palmitoylation inhibitors remains nascent, with their potential in dermatological diseases largely unexplored. This area therefore represents a promising frontier for future therapeutic discovery.

## 7. Conclusions and Perspective

Protein S-palmitoylation has been recognized for decades, and its roles in skin-associated diseases have increasingly attracted research interest. In an emerging field that lacks comprehensive reviews, this work addresses a clear gap in the literature. This review systematically summarizes the identified enzymes mediating S-palmitoylation and those responsible for its reversal. It also synthesizes current knowledge on their functions in dermatological conditions such as psoriasis and atopic dermatitis. Furthermore, the mechanistic underpinnings are discussed from multiple perspectives, encompassing cell differentiation, autophagy, ferroptosis and apoptosis. This work is expected to advance our understanding and accelerate the developing pace of some sub-disciplines in dermatosis. However, several key areas warrant future investigation: (1) Disordered immunological function has been recognized as the principal mechanism of several dermatosis, but the roles of S-palmitoylation on skin immunology remain poorly characterized. (2) While monoclonal antibodies targeting specific inflammatory factors (e.g., IL-4, IL-13, TNF-α) are clinically established, how S-palmitoylation regulates cytokine production requires elucidation. (3) Cutaneous infections represent a major dermatological concern, and S-palmitoylation may emerge as a potential mechanism of this problem inspired by S-palmitoylated GSDMD. (4) zDHHC13 is the most commonly identified zDHHC family protein in the pathogenesis of several dermatoses, but its therapeutic abilities in dermatosis remain unexplored. (5) The clinical translation of protein S-palmitoylation inhibitors represents a completely unexplored frontier in dermatology. This review has limitations, including a relatively superficial discussion of S-palmitoylation in inflammatory dermatoses and the unexplored potential for clinical translation in dermatology. Therefore, sustained research efforts, including high-quality mechanistic studies, are needed to advance this promising field.

## Figures and Tables

**Table 1:** Dermatologic phenotypes associated with protein S-palmitoylation.

Dermatologic Phenotypes	Enzymes	Substrates and Sites	Change of S-Palmitoylation	Pathogenic Consequence
Skin barrier	zDHHC13 [14,28]	Not identified [14,28]	Up	Promote skin barrier development
	zDHHC12 [30]	Claudin-3, Cys181/182/184 [30]	Up	Promote skin barrier integrity
Melanogenesis	zDHHC13 [32]	MC1R, Cys315 [32]	Up	Increase pigmentation, prevent melanoma occurrence
	zDHHC2,3 and 5 [15]	Tyrosinase, Cys500 [15]	Up	Diminish melanin content
	Not identified	Melanoregulin, N-terminus [29]	Up	Stabilize melanoregulin in the melanosome membrane
Alopecia	zDHHC13 [28]	Cornifelin, Cys58/59/60/95 [28]	Down	Cyclic alopecia
Atopic dermatitis	zDHHC13 [11]	Not identified	Down	Atopic dermatitis
Psoriasis	zDHHC2 [12]	Not identified	Up	Promote psoriasis progression
Skin inflammatory	zDHHC12 [36]	NLRP3, Cys 844 [36]	Up	Promote NLRP3 degradation
	zDHHC7 [31]	NLRP3, Cys 126 [31]	Up	Promote inflammasome assembly
	zDHHC5 [33]	NLRP3, Cys837/838 [33]	Up	Promote inflammasome activation
	zDHHC5 [34,35]	NOD1, Cys558/567/952 [34,35]	Up	Enhance NOD1’s stability
	zDHHC5 [34,35]	NOD2, Cys 395/1033 [34,35]	Up	Enhance NOD2’s stability
Carcinogenesis	zDHHC13 [37]	Not identified	Down	Inhibit malignant progression of papillomas

Illustrative examples of the identified roles and corresponsive substrates of S-palmitoylation mediators (zDHHCs) in dermatologic phenotypes.

**Table 2:** Associations between protein S-palmitoylation enzymes and physiological processes.

Physiological Processes	Enzymes	Substrate and Sites	Change of Palmitoylation
Differentiation	zDHHC13 [28]	Not identified	Up
Autophagy	zDHHC5 [58]	Beclin1 Cys137 [58]	Up
	zDHHC5 [40]	ATG16L1 Cys153 [40]	Up
	zDHHC1/11 [41]	MCOLN3/TRPML3, Cys549/550/551 [41]	Up Up
	PPT1 [42]	Rab7, Cys205/207 [42]	Up
Pyroptosis	zDHHC5 [47,48,49,50,59]	GSDMD, Cys 191/192 [47,48,49,50,59]	Up
	zDHHC7 [47,48,49,50,59]	GSDMD, Cys 191/192 [47,48,49,50,59]	Up
	zDHHC9 [47,48,49,50,59]	GSDMD, Cys 191/192 [47,48,49,50,59]	Up
	zDHHC14 [47,48,49,50,59]	GSDMD, Cys 191/192 [47,48,49,50,59]	Up
	Not identified	GSDMD, Cys 39,57 [52]	Up
	zDHHC 2/7/11/15 [53]	GSDME [53]	Up
Ferroptosis	DUXAP8 [44]	SCL7A11, Cys414 [44]	Up
	zDHHC8 [60]	SLC7A11, Cys327 [60]	Up
Apoptosis	zDHHC3 [45]	SLC9A2 [45]	Up

Illustrative examples of the identified roles and corresponsive substrates of S-palmitoylation mediators (zDHHCs) in physiological processes including cell differentiation, autophagy, pyroptosis, ferroptosis, and apoptosis.

**Table 3:** Associations between protein S-palmitoylation enzymes and organelles.

Organelles	Enzymes	Substrate and Sites	Direction of Palmitoylation Change
Mitochondria	zDHHC13 [61,69]	Drp1 [61]	UP
Endoplasmic reticulum	zDHHC9 [70]	BIP, Cys420 [70]	UP
Lysosome	Not identified	TMEM55B [71]	UP
	Not identified	Syt VII [65]	UP
	Not identified	IFNGR1, Cys122 [66]	UP
	Not identified	PD-1 [67]	UP

Illustrative examples of the identified roles and corresponsive substrates of S-palmitoylation mediators (zDHHCs) in organelles including mitochondria, endoplasmic reticulum and lysosomes.

**Table 4:** Chemical structure and mechanism of protein S-palmitoylation compounds.

Compound	Chemical Structure	Mechanism of S-Palmitoylation Inhibition	Selectivity
2-bromopalmitate (2-BP)	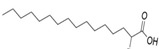	Forms a covalent bond with the cysteine in the DHHC motif on its α-position carbon [68]	Without
MY-D-2 (2-BP analog)	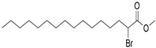	An electron-deficient ester increases reactivity of the α-position carbon [72]	Weak inhibiting potency against zDHHC 3/7
MY-D-3 (2-BP analog)	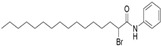	An electron-rich amide decreases reactivity of the α-position carbon [72]	Without obvious inhibiting potency
MY-D-4 (2-BP analog)	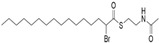	An electron-deficient thioester increases reactivity of the α-position carbon [72]	Strong inhibiting potency against zDHHC 3/7
MY-D-5 (MY-D-4 analog)	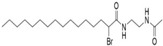	An additional electron-rich amide is added to decrease reactivity of the α-position carbon on MY-D-4 [72]	Weak inhibiting potency against zDHHC 3/7
MY-D-6 (MY-D-4 analog)	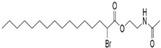	An additional electron-deficient ester is added to increase reactivity of the α-position carbon on MY-D-4 [72]	Weak inhibiting potency against zDHHC 3/7
Tetrazole-containing compound 1 (TTZ-1)	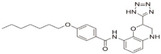	Inhibiting zDHHC 2 autoacylation [73]	zDHHC 2
Tetrazole-containing compound 2 (TTZ-2)	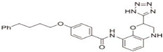	Inhibiting zDHHC 2 autoacylation [73]	zDHHC 2

Detailed information about the identified S-palmitoylation inhibitors and their chemical structures, mechanisms and selectivity.

## Data Availability

No new data were created or analyzed in this study.
